# Evidence-Based Crossword Puzzles for Health Professions Education: A Systematic Review

**DOI:** 10.1007/s40670-024-02085-x

**Published:** 2024-06-22

**Authors:** Matthew Arnold, Sheryn Tan, Tiani Pakos, Brandon Stretton, Joshua Kovoor, Aashray Gupta, Josephine Thomas, Stephen Bacchi

**Affiliations:** 1 University of Adelaide, Adelaide, SA 5005 Australia; 2 Royal Adelaide Hospital, Adelaide, SA 5000 Australia; 3 Gold Coast University Hospital, Southport, QLD 4215 Australia; 4 Flinders University, Bedford Park, SA 5042 Australia; 5 Northern Adelaide Local Health Network, Adelaide, SA Australia; 6 Ballarat Base Hospital, VIC Ballarat, 3350 Australia; 7 Lyell McEwin Hospital, Elizabeth Vale, SA 5112 Australia

**Keywords:** Gamification, medical education, enjoyment, engagement

## Abstract

Crossword puzzles have been utilised as a means of health professions education (HPE) gamification. A systematic review conducted in accordance with PRISMA guidelines was performed to evaluate the educational impact and describe the characteristics of crosswords in HPE contexts. Twenty-nine studies fulfilled inclusion criteria. Crossword puzzles are an enjoyable learning activity and provide positive educational impact. The available evidence suggests crossword puzzles increase student knowledge on objective measures.

**Supplementary Information:**

The online version contains supplementary material available at 10.1007/s40670-024-02085-x.

## Introduction

Health professions education (HPE), particularly in the setting of high-stakes standardised tests, may result in learners undertaking many practice questions. Gamification has been investigated as means by which to enhance the delivery of such content. Gamification involves “the application of elements of game playing (such as point scoring, competition with others, etc.) to other areas of activity, typically to encourage engagement” [1]. Studies in this area have provided promising results suggesting an increase in knowledge from educational games [2]. While the relative merits of gamification in medical education have been reviewed previously [3], different gamification styles present unique learning advantages and educators may benefit from nuanced information pertaining to the specific formats and delivery.

Crossword puzzles are word games in which numbered written clues or ‘stems’ are provided to the participant, and correspond to a grid in which the answers are written horizontally (across) or vertically (down), in a pattern such that shared letters intersect. Crossword puzzles are a widely used and familiar form of entertainment, appearing in newspapers, such as *The New York Times* since 1942 [4], and other popular periodicals. The linguistic morphology of crosswords may provide additional clues to answers beyond the information directly provided in the stem. These additional clues include the knowledge as to the number of letters in the answer, as well as any letters in the answer on the basis of previously solved stems for which answers intersect [5]. For HPE, crosswords can be used as a type of gamification to present information in a novel manner distinct from that of routine revision strategies, such as practice exam questions. The most effective means of delivery for the use of crosswords in HPE is unclear.

In view of this, we sought to answer the following question: in HPE students, what impact does the utilisation of crossword puzzles as a gamification strategy have on learning? In order to examine this question, we conducted a systematic review of the available literature. The primary aim of this study was to synthesise the evidence regarding the effectiveness of crosswords as a means of HPE. The secondary aim was to describe the characteristics of previously investigated crosswords (length, style, content & delivery) in the context of HPE.

## Methods

The development and reporting of this systematic review were in accordance with the Preferred Reporting Items for Systematic Reviews and Meta-Analyses (PRISMA) guidelines (see checklist in Supplementary Information) [6]. The protocol was registered prospectively with the PROSPERO registry (CRD42022378280). The databases PubMed, Embase and Cochrane Library were searched from database inception to 25 November 2022. Search terms included: (*crossword) AND (healthcare OR education OR teaching OR learning OR profession OR medical)*. Individual database search strings are available in Supplementary Information. Additionally, reference lists of included articles were searched for relevant studies.

Determination of whether studies met inclusion criteria was performed with a standardised form and in duplicate. Inclusion criteria were (1) Studies published in English; (2) Primary peer-reviewed research article (reviews and abstracts were excluded); (3) Delivered crossword puzzle(s) to individuals in a HPE pathway (including medical, nursing, pharmacy, and allied health, at the undergraduate or postgraduate level); (4) Presented data on the effectiveness of crosswords as a means of health profession education; and (5) Full-text of the article was available. Articles were screened for inclusion suitability based upon titles and abstracts. Studies that were likely to fulfil inclusion criteria, and in cases of uncertainty, were reviewed in full-text. Eligibility determination was conducted in duplicate (M.A., S.T., T.P., and S.B.). Instances of disagreement were resolved through discussion and consensus with a third author.

Data were extracted using a standardised spreadsheet, and included the following—participant characteristics: profession (e.g., medical, nursing, allied health), stage of training; study information: number of participants, response rate; crossword characteristics: topic/content (e.g., specifiers around anatomy, pharmacology, or specialty), length (i.e., number of rows/columns), stem style (e.g., question vs fill in the blank, full sentences vs sentence fragments), answers provided or not, circumstances of delivery (e.g., in teaching session vs during own time), undertaken individually or in group (or not specified), timing of delivery (e.g., relative to summative assessments); comparator characteristics (if relevant); and outcomes (educational impact, and student experience). The data are highlighted in the Supplementary Information. Methodological quality analysis and risk of bias assessment was performed using the Medical Education Research Study Quality Instrument (MERSQI) [7, 8]. The MERSQI tool was specifically designed for medical education contexts in which multiple study designs required appraisal, and considers the domains of study design, sampling, type of data, validity of evaluation, data analysis & outcomes [8]. Each domain is scored according to defined criteria, with a total score ranging from a minimum of 5 to maximum of 18, with a higher score indicating increasing methodological quality [8]. The MERSQI tool has been validated for use in medical education research [7], and is recommended for use in systematic reviews in medical education in an Association for Medical Education in Europe (AMEE) guide [9]. Methodological quality analysis utilising MERSQI was performed in duplicate with instances of disagreement resolved through discussion and consensus with a third investigator.

## Results

**Fig. 1:**
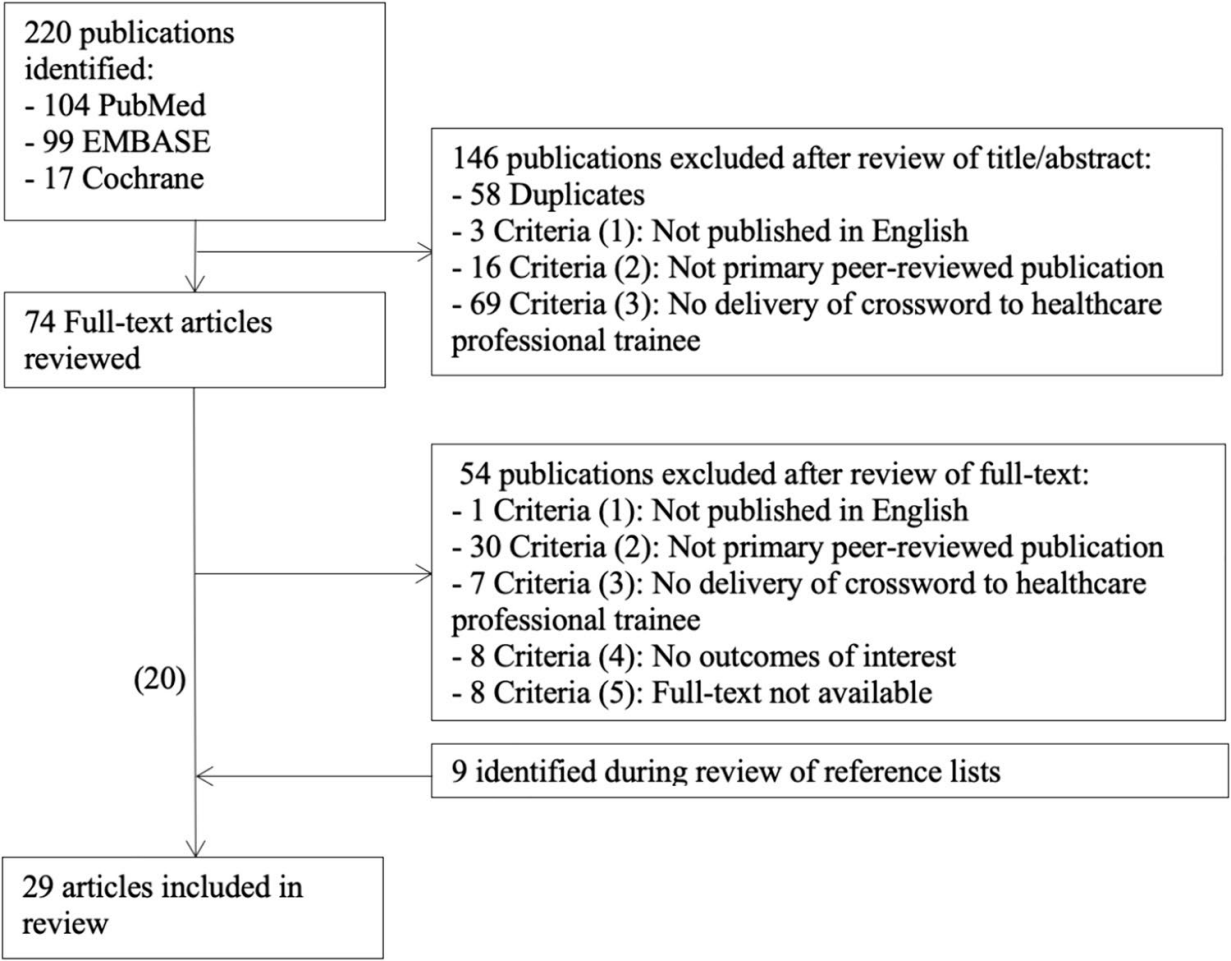
Flow diagram demonstrating study selection and exclusion rationale

Initial searches returned a total of 220 records. Of these, 29 fulfilled eligibility criteria and were included in the systematic review, with others excluded as indicated in the flow diagram provided in Fig. 1. Included studies were from a diverse array of countries, including 14 from India [10–23], 4 from the United States of America [24–27], 2 from Malaysia [28, 29], 2 from Saudi Arabia [30, 31], 2 from Oman [32, 33], 2 from Iran [34, 35], and one from each of Canada [36], the United Arab Emirates [37] and Palestine [38]. The sample size of the included studies ranged from 38 [10] to 425 [21]. The structure and delivery of crosswords employed varied substantially. Methodological quality varied with MERSQI scores ranging from 5.5 to 15.5 (mean score 10.1). The most common methodological limitations were studies being conducted at a single institution [10–38] and utilising only cross-sectional or post-test-only methodologies [10, 12–19, 21–33, 36, 37], and a number of studies also had unclear response rates [13, 15, 16, 19, 22, 24, 28, 30, 31, 34, 36, 37] (see Table 3, Supplementary Information D).

There were seven studies that report of the conduct of randomised trials [11, 12, 15, 20, 33, 35, 38]. Six examined student performance on knowledge-based assessments in the crossword groups compared to another group [11, 12, 15, 20, 35, 38]. In the study of speech therapy students by Zamani et al., both groups had similar performance prior to the intervention, and one month after the educational intervention the group that received crosswords had a significantly higher test score than the traditional teaching group (18.26 vs 16.10, P = 0.001) [35]. Similarly, in Gaikwad & Tankhiwale, the interventional group had an absolute learning gain of 33.9% as compared to the control group having an absolute learning gain of 18.55% (statistical significance not presented) [11]. These improvements in knowledge as evaluated through test scores were supported by other studies that examined trainees’ perceptions of their knowledge gain. For example, in Sannathimmappa et al. the proportion of students who "strongly agreed" with a statement that "Solving crossword puzzles improved my examination scores" was 69.3% [33]. Conversely, Shenoy & Rao compared crosswords to student led tutorials, and demonstrated improved test scores in the tutorial group, compared with that of the crossword group [20], though there was no control group who received traditional teaching methods to allow for comparison with standard practice. In the five studies that presented results regarding student experience, the responses were consistently positive with students reporting they enjoyed crosswords and would like to undertake further crosswords in future [11, 15, 20, 33, 35].

Eight studies fulfilled inclusion criteria that employed either single-group pre- and post-test analyses [16, 17, 19, 23, 24, 27, 37] or non-randomised two group studies [34]. Four studies presented data on the educational impact on test scores. Three studies demonstrated an increase in test scores following the application of crossword puzzles [23, 24, 34], noting that in one of these, crossword puzzles were not the sole intervention [24]. One study did not show an increase in test scores following crossword puzzle application [27]. Six studies presented results regarding student perceptions of educational effect, with all reporting that students felt that crossword puzzles had a positive impact on their learning [16, 17, 19, 23, 27, 37]. In the six studies that presented results regarding student experience, the responses were consistently positive in five [16, 17, 19, 23, 37], with one study reporting a range of responses from equivocal to positive [24].

There were 14 studies that conducted cross-sectional or post-test only evaluation [10, 13, 14, 18, 21, 22, 25, 26, 28–32, 36]. While these studies are generally of a lower quality of evidence to the previously described methodologies, the results of these studies were generally similar. The evidence that could be gleaned from these studies was typically more limited than those with randomised study designs. Only one study of this design reported the effect of crosswords on test performance, with that study reporting a positive effect in a cohort of learners in nursing programs [26]. The majority of these studies evaluated educational effect as reported by students. These reports were positive in all studies that evaluated such outcomes. The majority of trainees endorsed statements regarding the effect of crosswords on memory [10, 29], understanding [18, 29], and learning [13, 18, 29–32, 36]. The reported student experience was positive for the majority of participants in all studies of this type [10, 14, 18, 21, 25, 28–32, 36].

The length of examined crosswords varied substantially across the 19 studies which reported these characteristics [12, 16, 19, 23, 24, 27, 33, 37] ranging from 10 stems through to 60 stems, with the majority being 20 stems or fewer. Stems were most commonly presented as sentences or sentence fragments [10, 16, 22–25, 28, 30–33, 36, 37], with other methods including questions [24, 25] or fill-in the blanks [16, 23–25, 30, 32]. The majority of studies administered crosswords during teaching sessions [10–20, 22–25, 28–31, 33, 35–37], as opposed to in students’ own time [26, 32, 34, 38]. The majority also administered the crosswords in groups ranging from two to twelve students in size [12–14, 16–18, 20, 22, 24, 25, 27–29, 31, 32, 36, 37], with fewer studies administering crosswords individually [10, 11, 15, 19, 23, 30, 33, 35]. Several studies commented on the collaborative nature of crossword completion as a significant positive factor relating to enjoyment [14, 23, 25, 30, 36]. Notably, studies also reported that having a competitive element to crosswords facilitated learning [10, 17, 28, 32], and it is noted that these implementation strategies are not mutually exclusive. Crosswords were most commonly administered via printed paper copies [10–13, 15, 16, 19, 20, 22, 25, 29, 30, 33, 35–39]. Several studies did not clearly specify whether delivery was via printed or digital copies [17, 18, 23, 24, 26, 28, 31]. Two studies described fully digital crossword delivery, utilising a web-based platform [32] or an Android app [34], designed by the respective research teams. The remaining two studies described hybrid approaches, with one study [21] utilising a combination of printed copies in addition to Google Forms & Google Classroom platforms, and the other [14] providing electronic PDF documents which students could elect to either print or edit digitally.

## Discussion

This systematic review synthesised the available evidence, and found that crosswords have shown utility as an educational tool in a diverse array of learners, both with respect to HPE program and geography. Published studies have found generally positive results with respect to educational impact as evaluated through knowledge-based tests and student perceptions of knowledge gain. It was consistently reported that the majority of participants found crossword completion enjoyable. However, optimal crossword design for HPE has not been established. Knowledge of the ideal characteristics of crossword design can provide educators seeking to utilise this strategy with guidance as to how to develop these teaching materials. Examined crossword structures have included stems utilising sentences, sentence fragments, and fill-in-the-blank structures. The ideal method for crossword administration is uncertain, and is likely to vary based on local context. However, most studies have administered crosswords in group settings during teaching sessions. Participants have described collaborative completion as enjoyable, whilst also noting that the incorporation of competitive aspects to crosswords increased perceived effectiveness. Administration of crossword puzzles where learners compete in groups may therefore leverage the benefits of both strategies.

Efforts to make HPE more enjoyable may encourage the engagement and passion of learners, and have been explored through multiple avenues [40, 41]. It is evident that health professions students find crossword puzzles to be an enjoyable teaching method. However, it is relevant to consider the needs of learners at different stages. In the single included study (Dittus et al.) where the target group for crossword puzzles was practicing clinicians, the magnitude of overall satisfaction for the crossword puzzles themselves was reduced relative to traditional small group problem-based learning sessions, which may indicate less applicability of crosswords as a learning strategy with increasing levels of seniority [24]. The issues faced by practising health professionals in their daily work are likely to be of higher complexity, and thus, simpler education strategies such as crossword puzzles may be viewed as less relevant in this context, or there may be a perception that gamification is not a serious academic pursuit. However, the findings of this single study of 43 trainees should not be generalised too broadly without further research.

In evaluating outcomes, a modified Kirkpatrick four-level model can be utilised, which considers outcomes at the levels of reaction (satisfaction), learning (change in knowledge), behaviour and results (change in organizational practice) [42, 43]. The studies reviewed thus far have focussed on either reaction (level 1) or learning (level 2) based outcomes. Regarding level 1 outcomes on reactions, multiple studies reported that students subjectively judged crossword puzzles to have a positive impact on learning/educational outcomes, and felt that they should be included in courses/curriculum. Naturally, the subjective nature of these finding is an important limitation to consider. Furthermore, in the study by Sumanasekera et al. which compared crosswords with other active learning strategies, whilst 60–65% of respondents perceived that crossword puzzles helped retain concepts, only 6–7% of participants felt crosswords to be the most valuable learning method (with web-based interactive quizzes scoring the highest at 69–86%) [27]. Collaborative groupwork was described as contributing to the enjoyable nature of crosswords in multiple studies [14, 25, 30, 36]. Seemingly at odds with this result, several studies reported that students reported that a competitive aspect contributed to effectiveness [10, 17, 28, 32], although another noted more equivocal findings in this regard [37]. For medical students, there is other literature to support improved academic outcomes with competitive learning techniques [44]. However, a difference between professions may be a possible explanation, with a preference for collaboration in some cases, as two of the papers describing a benefit from collaboration involved pharmacy students [25, 30]. Further research could look further at the impact of crosswords in a competitive learning environment.

Regarding level 2 outcomes on learning [43], relatively fewer studies sought to assess the educational impact of crossword puzzles with regards to effects on knowledge as measured by objective test scores. Of those studies analysed, the majority indicate that crossword puzzles have a positive impact on student knowledge, though it is noted that this was not a unanimous finding [20, 27]. The study by Sumanasekera et al. did not demonstrate improved test scores following crossword puzzle administration, instead finding that videos and fill-in-the-blank tables were most effective in improving exam scores [27]. Further to this, Shenoy & Rao found that test scores were statistically significantly higher when learning was supported by student-led objective tutorials, compared with crossword puzzles [20]. These findings suggest a need for further high-quality research which compares crossword puzzles to other learning methods, looking specifically at objective measures of impact such as test scores.

This study has several limitations that should be acknowledged. Studies were limited to those published in English. Given the lexical nature of the topic of the review, the exclusion of non-English studies may limit the external generalisability of the findings for non-English speaking educators. Publication bias may have influenced the results of the review. The exclusion of an article due to the inability to retrieve such articles in full-text is also a limitation.

Future research in this area may also seek to examine the utility of crosswords at different stages of training. Such studies may be conducted comparing the utility of crosswords for junior medical students as compared to senior students, or postgraduate trainees. Additional studies examining different settings in which crosswords may be administered would also be useful. For example, no studies were identified that examined the use of crosswords for students specifically during clinical placements. Research examining the influence of different crossword structures (e.g., with respect to stem number) and designs (e.g., with respect to stem style, length & complexity) may also be useful, in order to determine if these variables impact upon learner reactions and outcomes from crossword administration. Such research should ideally seek to utilise robust randomised methodologies and evaluate effects on knowledge at least through the use of tests.

## Conclusion

These results demonstrate that crossword puzzles provide positive educational impact for learners in HPE contexts, particularly in terms of enjoyment. Learners find crossword puzzles to be an enjoyable learning activity, and have a positive perception regarding the impact on their learning. The available evidence suggests that crossword puzzles also have a positive educational impact as measured by knowledge-based assessments, though further research is warranted, given this finding was not unanimous, and was limited by methodological quality. The most commonly evaluated method of administration is in groups during teaching sessions. However, individual use and administration during students’ own time has also provided benefits. Further research may seek to examine variations of crosswords and crossword delivery to optimise potential educational gains.

## Supplementary Information

Below is the link to the electronic supplementary material.

Supplementary file1 (DOCX 81 KB)
