# Supplementary material for: Evidence-Based Crossword Puzzles for Health Professions Education: A Systematic Review
Source: Med Sci Educ. 2024 Jun 22;34(5):1231–7. doi: 10.1007/s40670-024-02085-x (PMC11496409; doi:10.1007/s40670-024-02085-x)
Supplement: Supplementary file 1 — Supplementary file1 (DOCX 81 KB) [file 40670_2024_2085_MOESM1_ESM.docx]

**Supplementary Information**

Supplementary Information Index:

1. Results tables
2. PRISMA checklist
3. Search terms
4. Risk of Bias Scoring utilising MERSQI

**Supplementary Information A: Results Tables**

Table 1 summarises the characteristics of the included studies themselves, including country of origin, study type and participant characteristics including sample size, health profession and stage of training.

**Table 1:** Study characteristics

| \|  \| **General** \| \| **Participant characteristics** \| \| \| \| \| --- \| --- \| --- \| --- \| --- \| --- \| --- \| \|  \| Country \| Type of study (according to MERSQI) \| Number of participants \| Training pathway of participants (e.g., medical, nursing) \| Stage of training of participants \| Response rate \| \| Agarwal et al. 2020[1] \| India \| Single-group cross-sectional or single-group posttest only \| 38 \| Forensic Medicine \| 5th semester \| 86.80% \| \| Hirkani et al 2022[2] \| India \| Single-group cross-sectional or single-group posttest only \| 250 \| Medicine \| 1st year \| 74.40% \| \| Chitturi et al. 2020[3] \| India \| Single-group cross-sectional or single-group posttest only \| 206 \| Medicine, physiotherapy, and nursing \| 2nd year \| Not specified \| \| Gupta et al. 2015[4] \| India \| Single-group cross-sectional or single-group posttest only \| 117 \| Medicine \| 8th and 9th semester \| Not specified \| \| Nazeer et al. 2018[5] \| India \| Single-group cross-sectional or single-group posttest only \| 150 \| Medicine \| 1st year \| 100% \| \| Singh et al. 2021[6] \| India \| Single-group cross-sectional or single-group posttest only \| 425 \| Medicine \| 5th semester \| 94.40% \| \| Htwe et al. 2012[7] \| Malaysia \| Single-group cross-sectional or single-group posttest only \| 193 \| Medicine \| Phase 2 MBBS \| Not specified \| \| Saran & Kumar 2015[8] \| Malaysia \| Single-group cross-sectional or single-group posttest only \| 70 \| Dentistry \| 2nd year \| 100% \| \| Bawazeer et al. 2022[9] \| Saudi Arabia \| Single-group cross-sectional or single-group posttest only \| 267 \| Pharmacy \| Not specified \| Not specified \| \| Agarwal et al. 2017[10] \| Saudi Arabia \| Single-group cross-sectional or single-group posttest only \| 200 \| Medicine, dentistry, undergraduate and postgraduate nursing \| Not specified \| Not specified \| \| Qutieshat et al. 2022[11] \| Oman \| Single-group cross-sectional or single-group posttest only \| 63 \| Dentistry \| 4th year \| 94% \| \| Shah et al. 2010[12] \| United States of America \| Single-group cross-sectional or single-group posttest only \| 144 \| Pharmacy \| Not specified \| 80% \| \| Torres et al. 2022[13] \| United States of America \| Single-group cross-sectional or single-group posttest only \| 102 \| Nursing \| Not specified \| 58.60% \| \| Saxena et al. 2009[14] \| Canada \| Single-group cross-sectional or single-group posttest only \| 80 \| Medicine \| 2nd year \| Not specified \| \| Malini et al. 2019[15] \| India \| Single-group pretest and posttest \| 150 \| Medicine \| 1st year \| 100% \| \| Patel & Dave 2019[16] \| India \| Single-group pretest and posttest \| 60 \| Medicine \| 1st year \| 100% \| \| Patrick et al. 2018[17] \| India \| Single-group pretest and posttest \| 139 \| Medicine \| 5th semester \| Not specified \| \| Kumar et al. 2015[18] \| India \| Single-group pretest and posttest \| 124 \| Medicine \| 1st year \| Not specified \| \| Bryant J 2016[19] \| United Arab Emirate \| Single-group pretest and posttest \| 99 \| Medicine \| 1st year \| Uncertain, but likely >75% \| \| Dittus et al. 2014[20] \| United States of America \| Single-group pretest and posttest \| 43 \| Medicine \| PGY-1, PGY-2, PGY-3 \| 33.0%-41.9% (not specified) \| \| Sumanasekera et al. 2020[21] \| United States of America \| Single-group pretest and posttest \| 79 \| Pharmacy \| 2nd year \| 50.60% \| \| Katebi et al. 2020[22] \| Iran \| Nonrandomized 2 group \| 40 \| Midwifery \| Not specified \| Not specified \| \| Gaikwad & Tankhiwale 2012[23] \| India \| Randomized controlled trial \| 70 \| Medicine \| 2nd year MBBS \| 100% \| \| Shenoy et al. 2021[24] \| India \| Randomized controlled trial \| 161 \| Medicine \| 2nd year \| 66.00% \| \| Gilani et al. 2020[25] \| India \| Randomized controlled trial \| 50 \| Dentistry \| Final year \| 100% \| \| Kolte et al. 2017[26] \| India \| Randomized controlled trial \| 78 \| Medicine \| 2nd year \| Not specified \| \| Shawahna & Jaber 2020[27] \| Palestine \| Randomized controlled trial \| 224 \| Nursing \| 2nd, 3rd or 4th year \| 85.71% \| \| Zamani et al. 2021[28] \| Iran \| Randomized controlled trial \| 83 \| Speech therapy \| 4th semester \| 100% \| \| Sannathimmappa et al. 2018[29] \| Oman \| Randomized controlled trial \| 114 \| Medicine \| 5th year \| 95% \| |
| --- | --- | --- | --- | --- | --- | --- | --- | --- | --- | --- | --- | --- | --- | --- | --- | --- | --- | --- | --- | --- | --- | --- | --- | --- | --- | --- | --- | --- | --- | --- | --- | --- | --- | --- | --- | --- | --- | --- | --- | --- | --- | --- | --- | --- | --- | --- | --- | --- | --- | --- | --- | --- | --- | --- | --- | --- | --- | --- | --- | --- | --- | --- | --- | --- | --- | --- | --- | --- | --- | --- | --- | --- | --- | --- | --- | --- | --- | --- | --- | --- | --- | --- | --- | --- | --- | --- | --- | --- | --- | --- | --- | --- | --- | --- | --- | --- | --- | --- | --- | --- | --- | --- | --- | --- | --- | --- | --- | --- | --- | --- | --- | --- | --- | --- | --- | --- | --- | --- | --- | --- | --- | --- | --- | --- | --- | --- | --- | --- | --- | --- | --- | --- | --- | --- | --- | --- | --- | --- | --- | --- | --- | --- | --- | --- | --- | --- | --- | --- | --- | --- | --- | --- | --- | --- | --- | --- | --- | --- | --- | --- | --- | --- | --- | --- | --- | --- | --- | --- | --- | --- | --- | --- | --- | --- | --- | --- | --- | --- | --- | --- | --- | --- | --- | --- | --- | --- | --- | --- | --- | --- | --- | --- | --- | --- | --- | --- | --- | --- | --- | --- | --- | --- | --- | --- | --- | --- | --- | --- | --- | --- | --- | --- | --- | --- | --- | --- | --- |

Table 2 summarises the characteristics of the crossword puzzles that were utilised in each study, focusing on structural elements including the length of the overall puzzle and the style of stems utilised, as well as the timing of the delivery of the crossword puzzles and whether learners completed them individually or with peers.

**Table 2:** Crossword characteristics and delivery

| \|  \| **Crossword characteristics** \| \| \| \| \| \| \| \| --- \| --- \| --- \| --- \| --- \| --- \| --- \| --- \| \|  \| Topic/content \| Length (i.e., number of stems) \| Stem style \| Answers provided \| Circumstances of delivery \| Undertaken individually or in group \| Timing of delivery relative to summative assessments \| \| Agarwal et al. 2020 \| Forensic Medicine \| 17 \| Sentences \| No \| Teaching session \| Individually \| Not specified \| \| Hirkani et al 2022 \| Physiology \| 50 \| Not specified \| Not specified \| Teaching session \| Group \| Not specified \| \| Chitturi et al. 2020 \| Medicine, physiotherapy, and nursing content respectively \| 10 \| Sentence fragments \| Not specified \| Teaching session \| Groups of 4-6 \| Not specified \| \| Gupta et al. 2015 \| Obstetrics and gynecology \| 50 \| Not specified \| Yes \| Teaching session \| Groups of 3-4 \| Not specified \| \| Nazeer et al. 2018 \| Anatomy \| 50 per set for 8 sets \| Not specified \| Yes \| Teaching session \| Groups of 6 \| Not specified \| \| Singh et al. 2021 \| Endocrinology (medications) \| 32 \| Not specified \| Not specified \| Not specified \| Not specified \| Not specified \| \| Htwe et al. 2012 \| Pathology \| 20 \| Sentences \| Not specified \| Teaching session \| Group of 8-10 students \| Not specified \| \| Saran & Kumar 2015 \| Not specified \| Not specified \| Not specified \| Yes \| Teaching session \| Group discussions were encouraged \| Not specified \| \| Bawazeer et al. 2022 \| Pharmacology \| 20 \| Sentences, sentence fragments, and fill-in-the-blanks. \| Yes \| Teaching session \| Individually \| Not specified \| \| Agarwal et al. 2017 \| Pathology (renal, haematology, gastrointestinal systems) \| Not specified \| Sentences \| Yes \| Teaching session \| Groups of 6-7 \| Not specified \| \| Qutieshat et al. 2022 \| Topic-specific and general dental terms \| 60 \| Sentences, sentence fragments, and fill-in-the-blanks. \| Not specified \| Own time \| Groups of 2-3 \| Not specified \| \| Shah et al. 2010 \| Pharmacology (gastrointestinal & anti-ulcer agents) \| 13 \| Sentences, fill in the blanks and questions \| Not specified \| Teaching session \| Groups \| Not specified \| \| Torres et al. 2022 \| Not specified \| Not specified \| Not specified \| Yes \| Own time \| Not specified \| Four days prior to cumulative quizzes \| \| Saxena et al. 2009 \| Haematology \| 26 \| Sentences and sentence fragments \| Not specified \| Teaching session \| Groups of 6-7 \| Not specified \| \| Malini et al. 2019 \| Endocrine physiology \| Not specified \| Not specified \| Not specified \| Teaching session \| Groups of 6 \| Not specified \| \| Patel & Dave 2019 \| Exercise physiology \| 19 \| Sentence fragments and fill in the blank \| Yes \| Teaching session \| Individually \| Not specified \| \| Patrick et al. 2018 \| Pharmacology (endocrine) \| 32 \| Not specified \| Not specified \| Teaching session \| Individually \| Not specified \| \| Kumar et al. 2015 \| Physiology \| 29 \| Sentence fragments and fill in the blanks \| Yes \| Teaching session \| Groups of 12 \| Not specified \| \| Bryant J 2016 \| Physiology (homeostasis) \| 15 \| Sentence fragments \| Yes \| Teaching session \| Groups of 7-8 \| Not specified \| \| Dittus et al. 2014 \| Internal medicine \| 16 \| Sentences, fill in the blanks and questions \| No \| Teaching session \| Group and against other teams \| Not specified \| \| Sumanasekera et al. 2020 \| Diabetes \| 20 \| Not specified \| Not specified \| In and out of classroom \| Groups of 2-3 \| Not specified \| \| Katebi et al. 2020 \| Clinical cases (Pregnancy, labour, and midwifery emergency) \| Not specified \| Not specified \| Yes \| Own time \| Not specified \| Not specified \| \| Gaikwad & Tankhiwale 2012 \| Pharmacology \| Not specified \| Not specified \| Not specified \| Teaching session \| individually \| Not specified \| \| Shenoy et al. 2021 \| Rheumatology \| Not specified \| Not specified \| Not specified \| Teaching session \| Groups of 5 \| Not specified \| \| Gilani et al. 2020 \| Orthodontics: (1) growth and development (2) development of dentition and occlusion \| 25-30 \| Not specified \| No \| Teaching session \| Groups of 3-4 \| Not specified \| \| Kolte et al. 2017 \| Pharmacology \| Not specified \| Not specified \| No \| Teaching session \| Individually \| Not specified \| \| Shawahna & Jaber 2020 \| Pharmacology (epilepsy) \| Not specified \| Not specified \| Not specified \| Own time \| Not specified \| Not specified \| \| Zamani et al. 2021 \| Not specified \| Not specified \| Cipher crossword \| Not specified \| Teaching sessions \| Group sessions, but undertook crosswords individually \| Not specified \| \| Sannathimmappa et al. 2018 \| Immunology and microbiology \| 12 \| Sentences \| Yes \| Teaching session \| Group session, but undertook crosswords individually \| Not specified \| |
| --- | --- | --- | --- | --- | --- | --- | --- | --- | --- | --- | --- | --- | --- | --- | --- | --- | --- | --- | --- | --- | --- | --- | --- | --- | --- | --- | --- | --- | --- | --- | --- | --- | --- | --- | --- | --- | --- | --- | --- | --- | --- | --- | --- | --- | --- | --- | --- | --- | --- | --- | --- | --- | --- | --- | --- | --- | --- | --- | --- | --- | --- | --- | --- | --- | --- | --- | --- | --- | --- | --- | --- | --- | --- | --- | --- | --- | --- | --- | --- | --- | --- | --- | --- | --- | --- | --- | --- | --- | --- | --- | --- | --- | --- | --- | --- | --- | --- | --- | --- | --- | --- | --- | --- | --- | --- | --- | --- | --- | --- | --- | --- | --- | --- | --- | --- | --- | --- | --- | --- | --- | --- | --- | --- | --- | --- | --- | --- | --- | --- | --- | --- | --- | --- | --- | --- | --- | --- | --- | --- | --- | --- | --- | --- | --- | --- | --- | --- | --- | --- | --- | --- | --- | --- | --- | --- | --- | --- | --- | --- | --- | --- | --- | --- | --- | --- | --- | --- | --- | --- | --- | --- | --- | --- | --- | --- | --- | --- | --- | --- | --- | --- | --- | --- | --- | --- | --- | --- | --- | --- | --- | --- | --- | --- | --- | --- | --- | --- | --- | --- | --- | --- | --- | --- | --- | --- | --- | --- | --- | --- | --- | --- | --- | --- | --- | --- | --- | --- | --- | --- | --- | --- | --- | --- | --- | --- | --- | --- | --- | --- | --- | --- | --- | --- | --- | --- | --- | --- | --- | --- | --- | --- | --- | --- | --- | --- | --- | --- | --- |

**Supplementary Information B: PRIMSA reporting guidelines**

The below checklist provides the page number location of each PRISMA specified item for systematic review (*n.b. page number locations by default refer to the full manuscript document, ‘SI’ denotes supplementary information document*).

| **Section and Topic** | **Item #** | **Checklist item** | **Location where item is reported** |
| --- | --- | --- | --- |
| **TITLE** | | |  |
| Title | 1 | Identify the report as a systematic review. | 1 |
| **ABSTRACT** | | |  |
| Abstract | 2 | See the PRISMA 2020 for Abstracts checklist. | 2 |
| **INTRODUCTION** | | |  |
| Rationale | 3 | Describe the rationale for the review in the context of existing knowledge. | 3 |
| Objectives | 4 | Provide an explicit statement of the objective(s) or question(s) the review addresses. | 3-4 |
| **METHODS** | | |  |
| Eligibility criteria | 5 | Specify the inclusion and exclusion criteria for the review and how studies were grouped for the syntheses. | 4 |
| Information sources | 6 | Specify all databases, registers, websites, organisations, reference lists and other sources searched or consulted to identify studies. Specify the date when each source was last searched or consulted. | 3 |
| Search strategy | 7 | Present the full search strategies for all databases, registers and websites, including any filters and limits used. | SI |
| Selection process | 8 | Specify the methods used to decide whether a study met the inclusion criteria of the review, including how many reviewers screened each record and each report retrieved, whether they worked independently, and if applicable, details of automation tools used in the process. | 4-5 |
| Data collection process | 9 | Specify the methods used to collect data from reports, including how many reviewers collected data from each report, whether they worked independently, any processes for obtaining or confirming data from study investigators, and if applicable, details of automation tools used in the process. | 4-5 |
| Data items | 10a | List and define all outcomes for which data were sought. Specify whether all results that were compatible with each outcome domain in each study were sought (e.g. for all measures, time points, analyses), and if not, the methods used to decide which results to collect. | 5-9, SI |
|  | 10b | List and define all other variables for which data were sought (e.g. participant and intervention characteristics, funding sources). Describe any assumptions made about any missing or unclear information. | 5-9, SI |
| Study risk of bias assessment | 11 | Specify the methods used to assess risk of bias in the included studies, including details of the tool(s) used, how many reviewers assessed each study and whether they worked independently, and if applicable, details of automation tools used in the process. | 5 |
| Effect measures | 12 | Specify for each outcome the effect measure(s) (e.g. risk ratio, mean difference) used in the synthesis or presentation of results. | N/A |
| Synthesis methods | 13a | Describe the processes used to decide which studies were eligible for each synthesis (e.g. tabulating the study intervention characteristics and comparing against the planned groups for each synthesis (item #5)). | N/A |
|  | 13b | Describe any methods required to prepare the data for presentation or synthesis, such as handling of missing summary statistics, or data conversions. | N/A |
|  | 13c | Describe any methods used to tabulate or visually display results of individual studies and syntheses. | N/A |
|  | 13d | Describe any methods used to synthesize results and provide a rationale for the choice(s). If meta-analysis was performed, describe the model(s), method(s) to identify the presence and extent of statistical heterogeneity, and software package(s) used. | N/A |
|  | 13e | Describe any methods used to explore possible causes of heterogeneity among study results (e.g. subgroup analysis, meta-regression). | N/A |
|  | 13f | Describe any sensitivity analyses conducted to assess robustness of the synthesized results. | N/A |
| Reporting bias assessment | 14 | Describe any methods used to assess risk of bias due to missing results in a synthesis (arising from reporting biases). | N/A |
| Certainty assessment | 15 | Describe any methods used to assess certainty (or confidence) in the body of evidence for an outcome. | N/A |
| **RESULTS** | | |  |
| Study selection | 16a | Describe the results of the search and selection process, from the number of records identified in the search to the number of studies included in the review, ideally using a flow diagram. | 6 |
|  | 16b | Cite studies that might appear to meet the inclusion criteria, but which were excluded, and explain why they were excluded. | N/A |
| Study characteristics | 17 | Cite each included study and present its characteristics. | SI |
| Risk of bias in studies | 18 | Present assessments of risk of bias for each included study. | SI |
| Results of individual studies | 19 | For all outcomes, present, for each study: (a) summary statistics for each group (where appropriate) and (b) an effect estimate and its precision (e.g. confidence/credible interval), ideally using structured tables or plots. | NA |
| Results of syntheses | 20a | For each synthesis, briefly summarise the characteristics and risk of bias among contributing studies. | NA |
|  | 20b | Present results of all statistical syntheses conducted. If meta-analysis was done, present for each the summary estimate and its precision (e.g. confidence/credible interval) and measures of statistical heterogeneity. If comparing groups, describe the direction of the effect. | NA |
|  | 20c | Present results of all investigations of possible causes of heterogeneity among study results. | NA |
|  | 20d | Present results of all sensitivity analyses conducted to assess the robustness of the synthesized results. | NA |
| Reporting biases | 21 | Present assessments of risk of bias due to missing results (arising from reporting biases) for each synthesis assessed. | NA |
| Certainty of evidence | 22 | Present assessments of certainty (or confidence) in the body of evidence for each outcome assessed. | NA |
| **DISCUSSION** | | |  |
| Discussion | 23a | Provide a general interpretation of the results in the context of other evidence. | 9-12 |
|  | 23b | Discuss any limitations of the evidence included in the review. | 9-12 |
|  | 23c | Discuss any limitations of the review processes used. | 9-12 |
|  | 23d | Discuss implications of the results for practice, policy, and future research. | 9-12 |
| **OTHER INFORMATION** | | |  |
| Registration and protocol | 24a | Provide registration information for the review, including register name and registration number, or state that the review was not registered. | 4 |
|  | 24b | Indicate where the review protocol can be accessed, or state that a protocol was not prepared. | 4-5 |
|  | 24c | Describe and explain any amendments to information provided at registration or in the protocol. | NA |
| Support | 25 | Describe sources of financial or non-financial support for the review, and the role of the funders or sponsors in the review. | 1 |
| Competing interests | 26 | Declare any competing interests of review authors. | 1 |
| Availability of data, code and other materials | 27 | Report which of the following are publicly available and where they can be found: template data collection forms; data extracted from included studies; data used for all analyses; analytic code; any other materials used in the review. | SI |

**Supplementary Information C: Individual Database Search Strings**

***Pubmed***

("crossword"[All Fields] OR "crosswords"[All Fields]) AND ("delivery of health care"[MeSH Terms] OR ("delivery"[All Fields] AND "health"[All Fields] AND "care"[All Fields]) OR "delivery of health care"[All Fields] OR "healthcare"[All Fields] OR "healthcare s"[All Fields] OR "healthcares"[All Fields] OR ("educability"[All Fields] OR "educable"[All Fields] OR "educates"[All Fields] OR "education"[MeSH Subheading] OR "education"[All Fields] OR "educational status"[MeSH Terms] OR ("educational"[All Fields] AND "status"[All Fields]) OR "educational status"[All Fields] OR "education"[MeSH Terms] OR "education s"[All Fields] OR "educational"[All Fields] OR "educative"[All Fields] OR "educator"[All Fields] OR "educator s"[All Fields] OR "educators"[All Fields] OR "teaching"[MeSH Terms] OR "teaching"[All Fields] OR "educate"[All Fields] OR "educated"[All Fields] OR "educating"[All Fields] OR "educations"[All Fields]) OR ("education"[MeSH Subheading] OR "education"[All Fields] OR "teaching"[All Fields] OR "teaching"[MeSH Terms] OR "teaches"[All Fields] OR "teach"[All Fields] OR "teachings"[All Fields] OR "teaching s"[All Fields]) OR ("learning"[MeSH Terms] OR "learning"[All Fields] OR "learn"[All Fields] OR "learned"[All Fields] OR "learning s"[All Fields] OR "learnings"[All Fields] OR "learns"[All Fields]) OR ("occupations"[MeSH Terms] OR "occupations"[All Fields] OR "profession"[All Fields] OR "professions"[All Fields] OR "profess"[All Fields] OR "professed"[All Fields] OR "professing"[All Fields] OR "profession s"[All Fields]) OR ("medic"[All Fields] OR "medical"[All Fields] OR "medicalization"[MeSH Terms] OR "medicalization"[All Fields] OR "medicalizations"[All Fields] OR "medicalize"[All Fields] OR "medicalized"[All Fields] OR "medicalizes"[All Fields] OR "medicalizing"[All Fields] OR "medically"[All Fields] OR "medicals"[All Fields] OR "medicated"[All Fields] OR "medication s"[All Fields] OR "medics"[All Fields] OR "pharmaceutical preparations"[MeSH Terms] OR ("pharmaceutical"[All Fields] AND "preparations"[All Fields]) OR "pharmaceutical preparations"[All Fields] OR "medication"[All Fields] OR "medications"[All Fields]))

***EMBASE***

(crossword and (healthcare or education or teaching or learning or profession or medical)).mp. [mp=title, abstract, heading word, drug trade name, original title, device manufacturer, drug manufacturer, device trade name, keyword heading word, floating subheading word, candidate term word]

***Cochrane library***

In Title Abstract Keyword: (crossword) AND (healthcare OR education OR teaching OR learning OR profession OR medical) - (Word variations have been searched)

**Supplementary Information D: Risk of bias of included studies utilising MERSQI**

Table 3 provides an overview of the MERSQI scores for each included article. Scoring was performed and recorded in accordance with the framework presented by Cook & Reed [30]. The possible score range is outlined in the row beneath each criterion heading, to enable analysis of the methodological quality of each aspect individually, which provides more detail beyond a single total score [30]. For each criterion, an increasing score represents an increase in methodological quality, with details on the full range of response options for each domain available in the aforementioned study [30] (*see specifically Table 1 on page 1069*).

**Table 3:** MERSQI Scoring

| \| **Study** \| **MERSQI Criteria & Score** \| \| \| \| \| \| \| \| \| \| --- \| --- \| --- \| --- \| --- \| --- \| --- \| --- \| --- \| --- \| \| **Study design** \| **Sampling: Number of Institutions** \| **Sampling: Response rate** \| **Type of data** \| **Validity of evaluation instrument** \| **Data analysis: sophistication** \| **Data analysis: appropriateness** \| **Outcomes** \| **Total** \| \| *(1 – 3)* \| *(0.5 – 1.5)* \| *(0.5 – 1.5)* \| *(1 or 3)* \| *(0 – 3)* \| *(1 – 2)* \| *(0 – 1)* \| *(1 – 3)* \| *(5 – 18)* \| \| **Agarwal et al. 2020** \| Single-group cross-sectional or single-group posttest only: 1 \| 1 institution: 0.5 \| < 50% or not reported: 0.5 \| Assessment by study participant: 1 \| 1 \| Descriptive analysis only: 1 \| Data analysis appropriate for study design and type of data: 1 \| Satisfaction attitudes perceptions opinions general facts: 1 \| 7 \| \| **Bawazeer et al. 2022** \| Single-group cross-sectional or single-group posttest only: 1 \| 1 institution: 0.5 \| 50%–74%: 1 \| Assessment by study participant: 1 \| 1 \| Beyond descriptive analysis: 2 \| Data analysis appropriate for study design and type of data: 1 \| Satisfaction attitudes perceptions opinions general facts: 1 \| 8.5 \| \| **Dittus et al. 2014** \| Single-group pretest and posttest: 1.5 \| 1 institution: 0.5 \| < 50% or not reported: 0.5 \| Objective: 3 \| 1 \| Descriptive analysis only: 1 \| Data analysis appropriate for study design and type of data: 1 \| Knowledge or skills: 1.5 \| 10 \| \| **Gaikwad et al. 2012** \| Randomized controlled trial: 3 \| 1 institution: 0.5 \| ≥ 75%: 1.5 \| Objective: 3 \| 2 \| Beyond descriptive analysis: 2 \| Data analysis appropriate for study design and type of data: 1 \| Knowledge or skills: 1.5 \| 14.5 \| \| **Hirkani et al. 2022** \| Single-group cross-sectional or single-group posttest only: 1 \| 1 institution: 0.5 \| 50%–74%: 1 \| Assessment by study participant: 1 \| 0 \| Descriptive analysis only: 1 \| Data analysis appropriate for study design and type of data: 1 \| Satisfaction attitudes perceptions opinions general facts: 1 \| 6.5 \| \| **Htwe et al. 2012** \| Single-group cross-sectional or single-group posttest only: 1 \| 1 institution: 0.5 \| < 50% or not reported: 0.5 \| Assessment by study participant: 1 \| 1 \| Descriptive analysis only: 1 \| Data analysis appropriate for study design and type of data: 1 \| Satisfaction attitudes perceptions opinions general facts: 1 \| 7 \| \| **Katebi et al. 2020** \| Nonrandomized 2 group: 2 \| 1 institution: 0.5 \| < 50% or not reported: 0.5 \| Objective: 3 \| 1 \| Beyond descriptive analysis: 2 \| Data analysis appropriate for study design and type of data: 1 \| Knowledge or skills: 1.5 \| 11.5 \| \| **Malini et al. 2019** \| Single-group pretest and posttest: 1.5 \| 1 institution: 0.5 \| ≥ 75%: 1.5 \| Objective: 3 \| 1 \| Beyond descriptive analysis: 2 \| Data analysis appropriate for study design and type of data: 1 \| Knowledge or skills: 1.5 \| 12 \| \| **Patel et al. 2019** \| Single-group pretest and posttest: 1.5 \| 1 institution: 0.5 \| ≥ 75%: 1.5 \| Objective: 3 \| 1 \| Descriptive analysis only: 1 \| Data analysis appropriate for study design and type of data: 1 \| Knowledge or skills: 1.5 \| 11 \| \| **Patrick et al. 2018** \| Single-group pretest and posttest: 1.5 \| 1 institution: 0.5 \| < 50% or not reported: 0.5 \| Objective: 3 \| 2 \| Descriptive analysis only: 1 \| Data analysis appropriate for study design and type of data: 1 \| Knowledge or skills: 1.5 \| 11 \| \| **Qutieshat et al. 2022** \| Single-group cross-sectional or single-group posttest only: 1 \| 1 institution: 0.5 \| ≥ 75%: 1.5 \| Assessment by study participant: 1 \| 1 \| Descriptive analysis only: 1 \| Data analysis appropriate for study design and type of data: 1 \| Satisfaction attitudes perceptions opinions general facts: 1 \| 8 \| \| **Chitturi et al. 2020** \| Single-group cross-sectional or single-group posttest only: 1 \| 1 institution: 0.5 \| < 50% or not reported: 0.5 \| Assessment by study participant: 1 \| 1 \| Descriptive analysis only: 1 \| Data analysis appropriate for study design and type of data: 1 \| Satisfaction attitudes perceptions opinions general facts: 1 \| 7 \| \| **Saxena et al. 2009** \| Single-group cross-sectional or single-group posttest only: 1 \| 1 institution: 0.5 \| < 50% or not reported: 0.5 \| Assessment by study participant: 1 \| 1 \| Descriptive analysis only: 1 \| Data analysis appropriate for study design and type of data: 1 \| Satisfaction attitudes perceptions opinions general facts: 1 \| 7 \| \| **Shah et al. 2010** \| Single-group cross-sectional or single-group posttest only: 1 \| 1 institution: 0.5 \| ≥ 75%: 1.5 \| Assessment by study participant: 1 \| 1 \| Descriptive analysis only: 1 \| Data analysis appropriate for study design and type of data: 1 \| Satisfaction attitudes perceptions opinions general facts: 1 \| 8 \| \| **Shawahna et al. 2020** \| Randomized controlled trial: 3 \| 1 institution: 0.5 \| ≥ 75%: 1.5 \| Objective: 3 \| 3 \| Beyond descriptive analysis: 2 \| Data analysis appropriate for study design and type of data: 1 \| Knowledge or skills: 1.5 \| 15.5 \| \| **Shenoy et al. 2021** \| Randomized controlled trial: 3 \| 1 institution: 0.5 \| ≥ 75%: 1.5 \| Objective: 3 \| 1 \| Beyond descriptive analysis: 2 \| Data analysis appropriate for study design and type of data: 1 \| Knowledge or skills: 1.5 \| 13.5 \| \| **Singh et al. 2021** \| Single-group cross-sectional or single-group posttest only: 1 \| 1 institution: 0.5 \| ≥ 75%: 1.5 \| Objective: 3 \| 1 \| Descriptive analysis only: 1 \| Data analysis appropriate for study design and type of data: 1 \| Knowledge or skills: 1.5 \| 10.5 \| \| **Sumanasekera et al. 2020** \| Single-group pretest and posttest: 1.5 \| 1 institution: 0.5 \| 50%–74%: 1 \| Objective: 3 \| 2 \| Beyond descriptive analysis: 2 \| Data analysis appropriate for study design and type of data: 1 \| Knowledge or skills: 1.5 \| 12.5 \| \| **Torres et al. 2022** \| Single-group cross-sectional or single-group posttest only: 1 \| 2 institutions: 1 \| 50%–74%: 1 \| Objective: 3 \| 1 \| Beyond descriptive analysis: 2 \| Data analysis appropriate for study design and type of data: 1 \| Knowledge or skills: 1.5 \| 11.5 \| \| **Zamani et al. 2021** \| Randomized controlled trial: 3 \| 1 institution: 0.5 \| ≥ 75%: 1.5 \| Objective: 3 \| 2 \| Beyond descriptive analysis: 2 \| Data analysis appropriate for study design and type of data: 1 \| Knowledge or skills: 1.5 \| 14.5 \| \| **Sannathimmappa et al. 2018** \| Randomized controlled trial: 3 \| 1 institution: 0.5 \| ≥ 75%: 1.5 \| Objective: 3 \| 2 \| Beyond descriptive analysis: 2 \| Data analysis appropriate for study design and type of data: 1 \| Knowledge or skills: 1.5 \| 14.5 \| \| **Kumar et al. 2015** \| Single-group pretest and posttest: 1.5 \| 1 institution: 0.5 \| < 50% or not reported: 0.5 \| Assessment by study participant: 1 \| 0 \| Descriptive analysis only: 1 \| Data analysis inappropriate for study design or type of data: 0 \| Satisfaction attitudes perceptions opinions general facts: 1 \| 5.5 \| \| **Agarwal & Rao 2017** \| Single-group cross-sectional or single-group posttest only: 1 \| 1 institution: 0.5 \| < 50% or not reported: 0.5 \| Assessment by study participant: 1 \| 0 \| Descriptive analysis only: 1 \| Data analysis appropriate for study design and type of data: 1 \| Satisfaction attitudes perceptions opinions general facts: 1 \| 6 \| \| **Gupta et al. 2015** \| Single-group cross-sectional or single-group posttest only: 1 \| 1 institution: 0.5 \| < 50% or not reported: 0.5 \| Assessment by study participant: 1 \| 1 \| Descriptive analysis only: 1 \| Data analysis appropriate for study design and type of data: 1 \| Satisfaction attitudes perceptions opinions general facts: 1 \| 7 \| \| **Gilani et al. 2020** \| Randomized controlled trial: 3 \| 1 institution: 0.5 \| ≥ 75%: 1.5 \| Objective: 3 \| 2 \| Beyond descriptive analysis: 2 \| Data analysis appropriate for study design and type of data: 1 \| Knowledge or skills: 1.5 \| 14.5 \| \| **Bryant 2016** \| Single-group pretest and posttest: 1.5 \| 1 institution: 0.5 \| ≥ 75%: 1.5 \| Assessment by study participant: 1 \| 1 \| Descriptive analysis only: 1 \| Data analysis appropriate for study design and type of data: 1 \| Satisfaction attitudes perceptions opinions general facts: 1 \| 8.5 \| \| **Kolte et al.** \| Randomized controlled trial: 3 \| 1 institution: 0.5 \| < 50% or not reported: 0.5 \| Objective: 3 \| 1 \| Beyond descriptive analysis: 2 \| Data analysis appropriate for study design and type of data: 1 \| Knowledge or skills: 1.5 \| 12.5 \| \| **Nazeer et al. 2018** \| Single-group cross-sectional or single-group posttest only: 1 \| 1 institution: 0.5 \| ≥ 75%: 1.5 \| Assessment by study participant: 1 \| 1 \| Descriptive analysis only: 1 \| Data analysis appropriate for study design and type of data: 1 \| Satisfaction attitudes perceptions opinions general facts: 1 \| 8 \| \| **Saran & Kumar 2015** \| Single-group cross-sectional or single-group posttest only: 1 \| 1 institution: 0.5 \| ≥ 75%: 1.5 \| Assessment by study participant: 1 \| 1 \| Descriptive analysis only: 1 \| Data analysis appropriate for study design and type of data: 1 \| Satisfaction attitudes perceptions opinions general facts: 1 \| 8 \| |
| --- | --- | --- | --- | --- | --- | --- | --- | --- | --- | --- | --- | --- | --- | --- | --- | --- | --- | --- | --- | --- | --- | --- | --- | --- | --- | --- | --- | --- | --- | --- | --- | --- | --- | --- | --- | --- | --- | --- | --- | --- | --- | --- | --- | --- | --- | --- | --- | --- | --- | --- | --- | --- | --- | --- | --- | --- | --- | --- | --- | --- | --- | --- | --- | --- | --- | --- | --- | --- | --- | --- | --- | --- | --- | --- | --- | --- | --- | --- | --- | --- | --- | --- | --- | --- | --- | --- | --- | --- | --- | --- | --- | --- | --- | --- | --- | --- | --- | --- | --- | --- | --- | --- | --- | --- | --- | --- | --- | --- | --- | --- | --- | --- | --- | --- | --- | --- | --- | --- | --- | --- | --- | --- | --- | --- | --- | --- | --- | --- | --- | --- | --- | --- | --- | --- | --- | --- | --- | --- | --- | --- | --- | --- | --- | --- | --- | --- | --- | --- | --- | --- | --- | --- | --- | --- | --- | --- | --- | --- | --- | --- | --- | --- | --- | --- | --- | --- | --- | --- | --- | --- | --- | --- | --- | --- | --- | --- | --- | --- | --- | --- | --- | --- | --- | --- | --- | --- | --- | --- | --- | --- | --- | --- | --- | --- | --- | --- | --- | --- | --- | --- | --- | --- | --- | --- | --- | --- | --- | --- | --- | --- | --- | --- | --- | --- | --- | --- | --- | --- | --- | --- | --- | --- | --- | --- | --- | --- | --- | --- | --- | --- | --- | --- | --- | --- | --- | --- | --- | --- | --- | --- | --- | --- | --- | --- | --- | --- | --- | --- | --- | --- | --- | --- | --- | --- | --- | --- | --- | --- | --- | --- | --- | --- | --- | --- | --- | --- | --- | --- | --- | --- | --- | --- | --- | --- | --- | --- | --- | --- | --- | --- | --- | --- | --- | --- | --- | --- | --- | --- | --- | --- | --- | --- | --- | --- | --- | --- | --- | --- | --- | --- | --- | --- | --- | --- | --- | --- | --- | --- | --- | --- | --- | --- | --- | --- | --- | --- | --- | --- |

**References**

1. Agarwal H, Singhal A, Yadav A. Crossword Puzzle: An Innovative Assessment Tool to Improve Learning of Students in Forensic Medicine. Medico-Legal Update. 2020;20:18-22.

2. Hirkani M, Hegde G, Kamath R, Sonwane T, Angane E, Gajbhiye R. Strategies to foster group cohesion in online learning environments: use of crossword and Hybrid Medical Pictionary. Advances in Physiology Education. 2022;46(1):30-4. doi: 10.1152/advan.00116.2021.

3. Chitturi R, Krupal V, Potti R, Inuganti R. A Study on Perception of Students Regarding Newer Teaching Methods in Medical Education. JOURNAL OF CLINICAL AND DIAGNOSTIC RESEARCH. 2020;14. doi: 10.7860/JCDR/2020/44221.13925.

4. Gupta U, Gupta N, Sinha P, Gupta S, Mahdi F. Creative Learning Using Crossword Puzzle as Learning Tool for Undergraduates in Obstetrics and Gynecology. Medical Education Online. 2015;J Contemp Med Edu, Online. doi: 10.5455/jcme.20150508051013.

5. Nazeer M, Sultana R, Ahmed M, Asad M, Sami W, Hattiwale H, et al. Crossword Puzzles as an Active Learning Mode for Student Directed Learning in Anatomy Teaching: Medical Undergraduate Perceptions. 2018.

6. Singh Matreja P, Kaur J, Yadav L. Acceptability of the use of crossword puzzles as an assessment method in Pharmacology. J Adv Med Educ Prof. 2021;9(3):154-9. doi: 10.30476/jamp.2021.90517.1413.

7. Htwe TT, Sabaridah I, Rajyaguru KM, Mazidah AM. Pathology crossword competition: an active and easy way of learning pathology in undergraduate medical education. Singapore Med J. 2012;53(2):121-3.

8. Saran R, Kumar S. Use of crossword puzzle as a teaching aid to facilitate active learning in dental materials Medical Science. 2015.

9. Bawazeer G, Sales I, Albogami H, Aldemerdash A, Mahmoud M, Aljohani MA, et al. Crossword puzzle as a learning tool to enhance learning about anticoagulant therapeutics. BMC Med Educ. 2022;22(1):267. doi: 10.1186/s12909-022-03348-0.

10. Agarwal A, Rao S. Creative Pathology Teaching With Word Puzzles Until Students Learn: A Study in a Medical University. Asian Journal of Research in Medical and Pharmaceutical Sciences. 2017;2:1-7. doi: 10.9734/AJRIMPS/2017/38416.

11. Qutieshat A, Al-Harthy N, Singh G, Chopra V, Aouididi R, Arfaoui R, et al. Interactive Crossword Puzzles as an Adjunct Tool in Teaching Undergraduate Dental Students. Int J Dent. 2022;2022:8385608. doi: 10.1155/2022/8385608.

12. Shah S, Lynch LM, Macias-Moriarity LZ. Crossword puzzles as a tool to enhance learning about anti-ulcer agents. Am J Pharm Educ. 2010;74(7):117. doi: 10.5688/aj7407117.

13. Torres ER, Williams PR, Kassahun-Yimer W, Gordy XZ. Crossword Puzzles and Knowledge Retention. J Eff Teach High Ed. 2022;5(1):18-29. doi: 10.36021/jethe.v5i1.244.

14. Saxena A, Nesbitt R, Pahwa P, Mills S. Crossword Puzzles Active Learning in Undergraduate Pathology and Medical Education. Archives of pathology & laboratory medicine. 2009;133(9):1457-62.

15. Malini M, Sudhir K, Narasimhaswamy N. Crossword puzzle as a tool to enhance learning among students in a medical school. National journal of physiology, pharmacy and pharmacology. 2019;9(8):1-797. doi: 10.5455/njppp.2019.9.0620304062019.

16. Patel J, Dave D. Implementation and evaluation of puzzle-based learning in the first MBBS students. National Journal of Physiology, Pharmacy and Pharmacology. 2019:1. doi: 10.5455/njppp.2019.9.0309628032019.

17. Patrick S, Vishwakarma K, Giri VP, Datta D, Kumawat P, Singh P, et al. The usefulness of crossword puzzle as a self-learning tool in pharmacology. J Adv Med Educ Prof. 2018;6(4):181-5.

18. Kumar L, Bangera S, Thalenjeri P. Introducing innovative crossword puzzles in undergraduate physiology teaching- learning process. Archives of Medicine and Health Sciences. 2015;3:127. doi: 10.4103/2321-4848.154964.

19. Bryant JD. Crossword Puzzles - Entertaining tool to reinforce lecture content in undergraduate physiology teaching. International journal of biomedical research. 2016;7:346-9.

20. Dittus C, Grover V, Panagopoulos G, Jhaveri K. Chief's seminar: turning interns into clinicians. F1000Res. 2014;3:213. doi: 10.12688/f1000research.5221.1.

21. Sumanasekera W, Turner C, Ly K, Hoang P, Jent T, Sumanasekera T. Evaluation of multiple active learning strategies in a pharmacology course. Currents in Pharmacy Teaching and Learning. 2020;12. doi: 10.1016/j.cptl.2019.10.016.

22. Katebi MS, Leilimosalanejad, Bazrafkan L. Development of midwifery emergency curriculum by the clinical case-based crossword games simulation and learning in midwifery students. Pakistan Journal of Medical and Health Sciences. 2020;14:1126-30.

23. Gaikwad N, Tankhiwale S. Crossword puzzles: self-learning tool in pharmacology. Perspect Med Educ. 2012;1(5-6):237-48. doi: 10.1007/s40037-012-0033-0.

24. Shenoy D, Rao D. Crossword puzzles versus Student-Led Objective Tutorials (SLOT) as innovative pedagogies in undergraduate medical education. Scientia Medica. 2021;31:e37105. doi: 10.15448/1980-6108.2021.1.37105.

25. Gilani R, Niranjane P, Daigavane P, Bajaj P, Mankar N, Vishnani R. Crossword puzzle: An effective self-learning modality for dental undergraduates. Journal of Datta Meghe Institute of Medical Sciences University. 2020;15(3):397-401. doi: 10.4103/jdmimsu.jdmimsu_233_20.

26. Kolte S, Jadhav PR, Deshmukh YA, Patil A. Effectiveness of crossword puzzle as an adjunct tool for active learning and critical thinking in Pharmacology. International Journal of Basic & Clinical Pharmacology. 2017;6(6). doi: 10.18203/2319-2003.ijbcp20172236.

27. Shawahna R, Jaber M. Crossword puzzles improve learning of Palestinian nursing students about pharmacology of epilepsy: Results of a randomized controlled study. Epilepsy Behav. 2020;106:107024. doi: 10.1016/j.yebeh.2020.107024.

28. Zamani P, Biparva Haghighi S, Ravanbakhsh M. The use of crossword puzzles as an educational tool. J Adv Med Educ Prof. 2021;9(2):102-8. doi: 10.30476/jamp.2021.87911.1330.

29. Sannathimmappa M, Nambiar V, Gowda S, Arvindakshan R. Crossword puzzle: a tool for enhancing medical students' learning in microbiology and immunology. International Journal of Research in Medical Sciences. 2018;6:756. doi: 10.18203/2320-6012.ijrms20180591.

30. Cook DA, Reed DA. Appraising the quality of medical education research methods: the Medical Education Research Study Quality Instrument and the Newcastle-Ottawa Scale-Education. Acad Med. 2015;90(8):1067-76. doi: 10.1097/acm.0000000000000786.
